# Glycosaminoglycan Interactions in Murine Gammaherpesvirus-68 Infection

**DOI:** 10.1371/journal.pone.0000347

**Published:** 2007-04-04

**Authors:** Laurent Gillet, Heiko Adler, Philip G. Stevenson

**Affiliations:** 1 Division of Virology, Department of Pathology, University of Cambridge, Cambridge, United Kingdom; 2 GSF-Research Center for Environment and Health, Institute of Molecular Immunology, Clinical Cooperation Group Hematopoietic Cell Transplantation, Munich, Germany; Institut Pasteur, France

## Abstract

Glycosaminoglycans (GAGs) commonly participate in herpesvirus entry. They are thought to provide a reversible attachment to cells that promotes subsequent receptor binding. Murine gamma-herpesvirus-68 (MHV-68) infection of fibroblasts and epithelial cells is highly GAG-dependent. This is a function of the viral gp150, in that gp150-deficient mutants are much less GAG-dependent than wild-type. Here we show that the major MHV-68 GAG-binding protein is not gp150 but gp70, a product of ORF4. Surprisingly, ORF4-deficient MHV-68 showed normal cell binding and was more sensitive than wild-type to inhibition by soluble heparin rather than less. Thus, the most obvious viral GAG interaction made little direct contribution to infection. Indeed, a large fraction of the virion gp70 had its GAG-binding domain removed by post-translational cleavage. ORF4 may therefore act mainly to absorb soluble GAGs and prevent them from engaging gp150 prematurely. In contrast to gp70, gp150 bound poorly to GAGs, implying that it provides little in the way of adhesion. We hypothesize that it acts instead as a GAG-sensitive switch that selectively activates MHV-68 entry at cell surfaces.

## Introduction

Glycosaminoglycans (GAGs) are ubiquitous components of mammalian epithelial surfaces [1], and many viruses exploit them as entry co-factors. GAG binding is thought to allow virions to roll on cell surfaces until they encounter a protein ligand [2]. Herpesviruses encode a particular abundance of GAG binding proteins. For example, the Herpes simplex virus (HSV) glycoproteins B (gB), C and D all bind to GAGs [3]–[5], as do the Kaposi's Sarcoma-associated Herpesvirus (KSHV) gB, ORF4 and K8.1 [6]–[9]. Why these viruses should have so many GAG-binding proteins is unclear. Each also has several cell surface protein ligands, so the additional adhesion conferred by GAG binding would seem to be superfluous. This puzzle over GAGs is part of a more general conceptual challenge that the extraordinarily elaborate entry of herpesviruses presents. While most viruses make do with a single cell-binding glycoprotein, herpesviruses typically employ at least four. Since herpesviruses are generally transmitted from immune hosts, the obvious explanation for this complexity would be antibody evasion.

GAGs have received relatively little attention in gamma-herpesvirus infections because the archetypal gamma-herpesvirus, Epstein-Barr virus (EBV), appears to ignore them [10]. However, several gamma-2-herpesviruses do use GAGs for entry. Besides KSHV, GAG binding has been reported for the Bovine herpesvirus-4 (BHV-4) gB [11] and the Herpesvirus saimiri ORF51 [12]. The narrow species tropisms of these viruses have somewhat limited our understanding of how they work. Thus, the small animal model provided by the murine gammaherpesvirus-68 (MHV-68) [13], [14] provides an important experimental tool. MHV-68 naturally infects yellow-necked mice [15] and appears also to behave as a natural pathogen of inbred laboratory strains, in that it persists without causing disease unless there is immune suppression. At least 90% of its genes have clear homologs in KSHV or EBV [16]. In order to derive the greatest benefit from MHV-68 pathogenesis data, it is necessary to identify the protein functions that lie behind them. For example, MHV-68 infects epithelial cells, fibroblasts, B cells [17], macrophages [18] and dendritic cells [19], but understanding this tropism requires an understanding of the MHV-68 glycoproteins. Here we have addressed how these glycoproteins interact with GAGs.

MHV-68 infection of fibroblasts is highly sensitive to inhibition by soluble heparin [20]. We have previously characterized this sensitivity as a function of gp150, the MHV-68 M7 gene product. Thus, when M7 is disrupted infection is much less GAG-dependent. However, gp150, in contrast to its KSHV and Herpesvirus saimiri homologs, has not been shown to bind to GAGs. Nor has GAG binding been demonstrated for any other MHV-68 gene product. In this paper, we identify the major GAG-binding protein of MHV-68 as gp70, a product of ORF4, previously defined as a complement control protein [21], [22]. Surprisingly, gp70-deficient MHV-68 showed no obvious infection deficit and remained at least as GAG-dependent as wild-type. In contrast, gp150-deficient MHV-68 showed a marked, GAG-related phenotype but an association of gp150 with GAGs was hard to find. We found no evidence for the MHV-68 gB binding to GAGs, even though it has an equivalent “GAG-binding” motif to KSHV and BHV-4. We propose a model in which MHV-68 uses GAGs as a cell surface-associated entry cue via gp150 rather than for adhesion via gp70. The rationale is that MHV-68 can thereby minimise the exposure of key glycoprotein epitopes to antibody.

## Results

### ORF4-specific monoclonal antibodies

**Figure 1:**
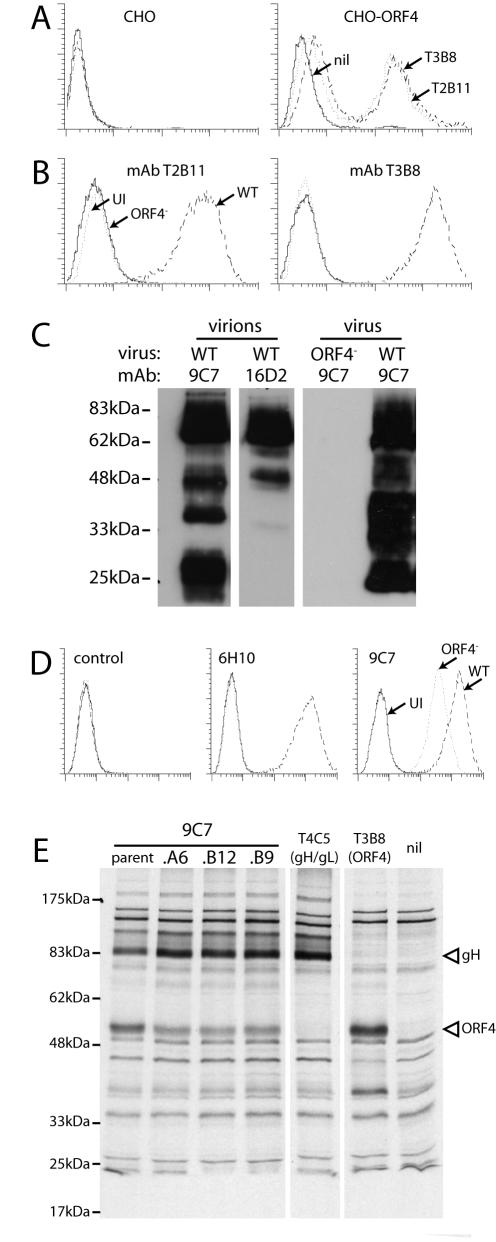
Identification and characterization of mAbs specific for MHV-68 ORF4 gene products. A. MAbs from MHV-68-infected mice were used to stain either untransfected CHO-K1 cells (CHO) or CHO-K1 cells transfected with an ORF4 expression construct (CHO-ORF4). These cells were not cloned. Approximately 50% of them appeared to express ORF4. All ORF4-specific mAbs gave equivalent staining. T3B8 and T2B11 are shown as examples. nil = secondary antibody only. B. BHK-21 cells were either left uninfected (UI) or infected (18 h, 2 PFU/cell) with wild-type (WT) or ORF4-deficient (ORF4^−^) MHV-68. They were then trypsinized and analyzed for mAb binding by flow cytometry. Again, mAbs T2B11 and T3B8 are shown as representative examples. All ORF4-specific mAbs gave similar results except 9C7 (see D). C. Wild-type (WT) or ORF4-deficient (ORF4^−^) viruses were directly recovered from infected cell supernatants (virus) or also purified on density gradients (virions), denatured, resolved by SDS-PAGE and immunoblotted with mAbs 9C7 or 16D2. D. BHK-21 cells were left uninfected (UI) or infected with wild-type (WT) or ORF4-deficient (ORF4^−^) MHV-68 as in B, followed by flow cytometric analysis of mAb binding. E. BHK-21 cells were infected (18 h, 2 PFU/cell) with wild-type MHV-68, then labelled for 1 h with ^35^S-cysteine/methionine. Viral proteins were immunoprecipitated from cell lysates with mAbs as indicated plus protein A-sepharose. A6, B12 and B9 are subclones of 9C7. T4C5 is a gH/gL-specific mAb. nil = protein A-sepharose only. The bands corresponding to gH and ORF4 product are indicated.

Our aim was to understand the GAG dependence of MHV-68 in the context of its GAG-binding glycoproteins. Our starting hypothesis was that gp150, ORF4 and gB could all have functionally important GAG interactions. We have previously characterized monoclonal antibodies (mAbs) for the identification of gp150 [20] and gB [23]. We completed this set of reagents by characterizing 5 mAbs that recognize ORF4 (Table 1). All were derived from MHV-68-infected mice. With the exception of 9C7 (see below), they recognized both CHO cells transfected with an ORF4 expression vector (Figure 1A) and BHK-21 cells infected with wild-type but not ORF4-deficient MHV-68 (Figure 1B). MAbs T2B11 and T3B8 are shown as examples. MAbs 16D2 and 9C7 recognized ORF4 products in immunoblots of purified virions (Figure 1C). The major band recognized by 16D2 had an apparent molecular weight of 70 kDa (gp70), equivalent to the 60-65 kDa band described by Kapadia et al. [21]. 9C7 recognized both this band and one of 25 kDa (gp25).

**Table 1:** ORF4-specific mAbs used in this study.

mAb	isotype	target^1^
T3B8	IgG1	SCR.4
T2B11	IgM	SCR.1
6H10	IgG2a	SCR.1
16D2	IgG2a	SCR.4
9C7	IgG2b	ST

1 The predicted ORF4 gene product is a type I transmembrane glycoprotein with 4 short consensus repeat (SCR) domains, followed by an ST-rich domain, a transmembrane domain and a short cytoplasmic tail.

**Figure 2:**
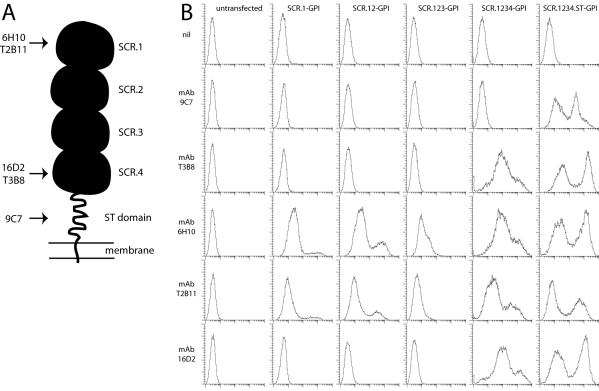
Mapping the ORF4-specific mAb targets. A. A schematic diagram of ORF4 with its 4 SCR domains, ST domain and membrane anchor, summarizing the information from B on approximate mAb binding sites. B. 293T cells were transfected with GPI-linked ORF4 truncations. 48 h later, the cells were trypsinized and analyzed for mAb binding by flow cytometry. nil = secondary antibody only.

MAb 9C7 recognized transfected ORF4 much like T3B8 and T2B11 in Figure 1A (data not shown) but still stained cells infected with ORF4-deficient MHV-68, albeit at a lower level than those infected with wild-type (Figure 1D). Despite rigorous subcloning, 9C7 immunoprecipitated both ORF4 and gH (Figure 1E). These proteins were not associated in the lysates, because other mAbs precipitated them separately (Figure 1E). 9C7 also recognized transfected gH/gL (data not shown). Other mAbs recognizing an overlapping gH/gL epitope [13] failed to recognize ORF4 (data not shown) and the 9C7 immunoblot signal was entirely ORF4-specific (Figure 1C). Thus, 9C7 recognized 2 distinct MHV-68 glycoprotein epitopes, presumably via different interactions. Despite this complication, it still proved useful in understanding ORF4 processing (Figure 2).

### Mapping ORF4 mAb recognition

We used GPI-linked ORF4 truncations to map the domains recognized by each mAb (Figure 2). The predicted ORF4 gene product is a type I glycoprotein with an extracellular domain comprising 4 short consensus repeats (SCRs) followed by an ST-rich segment (Figure 2A) [17]. SCR1 was sufficient for recognition by mAbs T2B11 and 6H10; mAbs 16D2 and T3B8 required SCR4; 9C7 required the ST domain (Figure 2B). (Whether the more N-terminal SCRs were also required for recognition was not determined.) Thus, the gp25 product recognized by 9C7 but not 16D2 (Figure 1C) seemed likely to be a C-terminal fragment of gp70, including its transmembrane and ST domains but missing SCR4. This fragment was presumably generated by post-translational cleavage, since RT-PCR showed no evidence of ORF4 splicing (data not shown).

### Soluble and membrane bound ORF4 products

**Figure 3:**
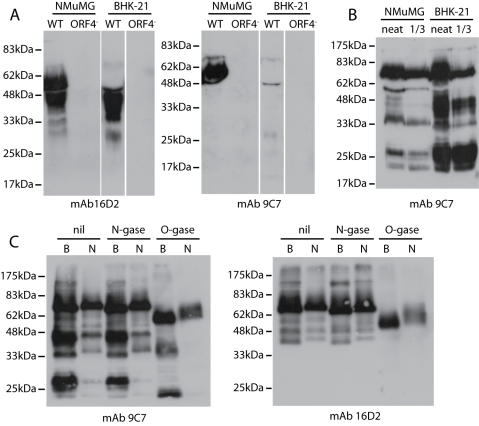
Identification of ORF4 gene products. A. NMuMG epithelial cells or BHK-21 fibroblasts were infected with wild-type (WT) or ORF4-deficient (ORF4^−^) MHV-68 (2 PFU/cell). 48 h later, cells were removed by low speed centrifugation (400×g, 10 min) and virions then removed by high speed centrifugation (20,000×g, 3 h). Supernatants were immunoblotted with mAbs 16D2 or 9C7. B. The wild-type virions from A were similarly analyzed by SDS-PAGE and immunoblotting. Neat and 1/3 diluted lysates are shown. C. Virus from BHK-21 (B) or NMuMG cells (N) was denatured and then left undigested (nil) or treated with PNGase F (N-gase) or sialidase plus O-glycanase (O-gase). All samples were resolved by SDS-PAGE and immunoblotted with mAbs 9C7 or 16D2.

An uncharacterized 40-45 kDa ORF4 product has been detected in 50×concentrated supernatants of MHV-68-infected cells [21]. MAb 16D2 readily detected a 45 kDa ORF4 gene product in the unconcentrated supernatants of MHV-68-infected BHK-21 cells (Figure 3A). MAb 9C7 did not detect this product (Figure 3A), implying that it included SCR4 but not the ST domain. Thus, it appeared that BHK-21 cells cleave gp70 between SCR4 and the ST domain to shed a 45 kDa SCR1-4 fragment and leave the 25 kDa ST domain in the virion membrane. Judging by 9C7 immunoblots (Figure 1B), most of the virion gp70 was cleaved in this way.

MHV-68-infected NMuMG cells showed a slightly different pattern, releasing ORF4 products of both 45 kDa and 55 kDa (Figure 3A). The 55 kDa form was bound by mAb 9C7, implying that it was generated from more C-terminal gp70 cleavage site than the 45 kDa form. NMuMG cell-derived virions correspondingly contained less gp25 (Figure 3B). Thus, NMuMG cells could cleave gp70 in either of 2 sites to release SCRs1-4. The minor 30-60 kDa, 9C7-reactive bands in BHK-21 cell-derived virions (Figure 1C, 3B) and 30-40 kDa, 16D2-reactive bands in BHK-21 cell supernatants (Figure 3A) may be the products of more N-terminal cleavages. So while KSHV generates a soluble form of ORF4 by alternative splicing [24], MHV-68 achieves the same end - release of the SCR domains-by post-translational cleavage.

The gp70 ST domain is predicted to be heavily O-glycosylated. We analyzed the contribution of glycans to the ORF4 apparent size by treating virus lysates with PNGase F to remove N-linked glycans or with sialidase plus O-glycanase to remove common O-linked glycans (Figure 3C). O-glycans evidently made a substantial contribution to the sizes of gp70 and gp25, whereas N-linked glycans did not. The predicted molecular weight of the full-length, unglycosylated ORF4 product is 40 kDa, so O-linked glycans, predominantly in the ST domain (Figure 3C), contributed approximately 30 kDa.

### ORF4 encodes the major MHV-68 GAG-binding glycoprotein

**Figure 4:**
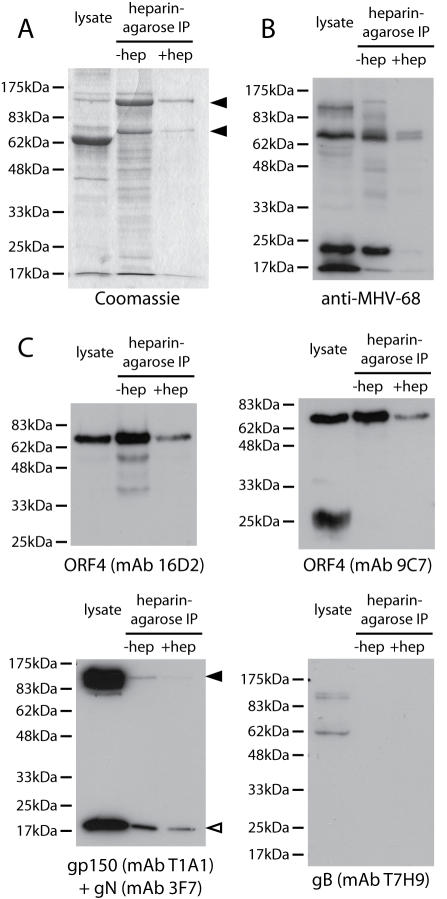
Identification of MHV-68 heparin binding proteins by heparin-agarose immunoprecipitation. A. Virus from BHK-21 cells was lysed in 1% Triton X-100 (lysate). 10% fetal calf serum is added to stabilize the virus, so albumin appears as a prominent 63 kDa band in the lysate. Pull-downs with heparin-agarose beads alone (-hep) or with additional 1 mg/ml soluble heparin (+ hep) were resolved by SDS-PAGE and stained with Coomassie R250. The prominent 150 kDa and 70 kDa viral bands are indicated. B. The same samples were immunoblotted with a rabbit serum raised against whole virus. At this exposure, strong signals are seen for only the most immunogenic viral proteins. C. The same samples were immunoblotted for ORF4 with mAbs 16D2 and 9C7, and for gp150, gN and gB with mAbs T1A1, 3F7 and T7H9. T1A1 and 3F7 were combined in one immunoblot because their targets are readily distinguished. The filled arrow marks gp150 and the open arrow gN.

Since heparin inhibits MHV-68 infection of GAG-expressing cells [20], we looked for evidence of GAG binding by gp70, gp150 or gB by heparin-agarose pull-downs of virion lysates (Figure 4A). Coomassie staining identified prominent bands of 150 kDa and 70 kDa. The 63 kDa lysate band is bovine albumin, which does not bind to heparin and therefore provides a control of immunoprecipitation specificity. Specificity was further confirmed by inhibiting precipitation with soluble heparin (Figure 4A). An MHV-68-immune rabbit serum (Figure 4B) strongly recognized the 70 kDa band and a 20 kDa band, but not the 150 kDa band. The 70 kDa band was confirmed as gp70 by immunoblotting with mAbs 16D2 and 9C7 (Figure 4C). Gp25 was not detected. It therefore did not appear to contain the gp70 heparin binding domain.

The immune rabbit serum used strongly recognizes gp150 (as can be seen in the lysate lane of Figure 4B), so it seemed unlikely that the 150 kDa band was gp150. This suspicion was confirmed by immunoblotting for gp150 (Figure 4C), which showed little signal in precipitates compared to lysates. We observed a similar minor recovery of gN, probably due to the precipitation of residual, non-disrupted virions. In contrast, to these trace amounts, gp70 was enriched in precipitates compared to lysates. gB was not enriched in precipitates (Figure 4C).

We identified the precipitated 150 kDa band by mass spectrometry. Its recovered peptides gave 67% coverage of ORF75c and 14% coverage of ORF75b. These ORFs are predicted to encode tegument proteins that would not be accessible to heparin in intact virions. The 20 kDa band was identified as the ORF65 capsid component (67% coverage). The association of the ORF75c, ORF75b and ORF65 gene products with heparin presumably reflected that they normally interact with another negatively charged polymer such as DNA. The only glycoprotein that bound convincingly to heparin-agarose was gp70.

### The N-terminal SCRs of ORF4 bind to GAGs

**Figure 5:**
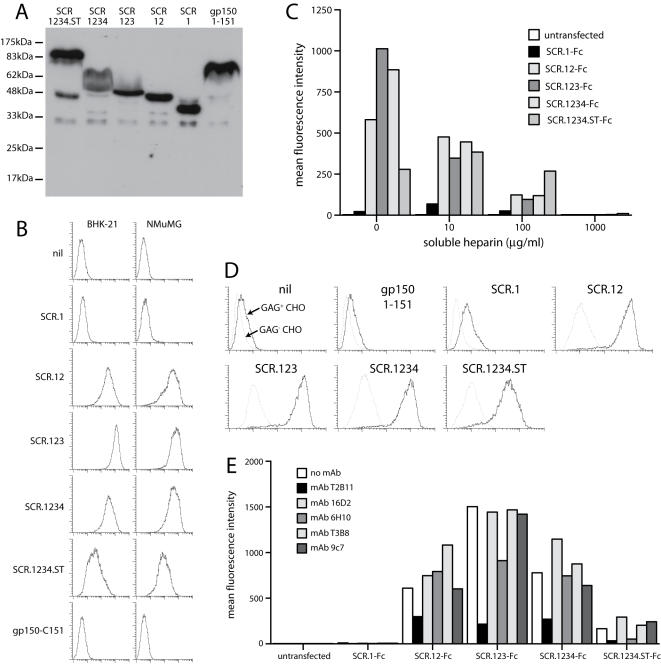
Heparin binding by ORF4 gene products. A. Gp70 truncations were fused to C-terminal human IgG_1_ Fc and expressed by transient transfection of 293T cells. An equivalent fusion of gp150 amino acids 1-151 was used as a control. 293T cell supernatants were immunoblotted for fusion protein content with a human IgG Fc-specific pAb. B. The same supernatants were used to stain unfixed BHK-21 cells or NMuMG cells. Protein binding was assayed by flow cytometry. nil = supernatant from untransfected cells. 1 of 3 equivalent experiments is shown. C. The binding of each fusion protein to L929 cells was tested with or without soluble heparin. The fusion proteins were pre-incubated with heparin (1 h, 4°C) and then added to the cells. The graph summarizes data similar to those shown in B, from 1 of 3 equivalent experiments, each acquiring 30,000 events per sample. D. The binding of each Fc fusion protein to GAG^+^ or GAG^−^ CHO cells was assayed by flow cytometry. 1 of 2 equivalent experiments is shown. E. Each ORF4-specific mAb was tested for its capacity to inhibit BHK-21 cell binding by each Fc fusion protein. The mAbs and Fc fusion proteins were incubated together (1 h, 4°C) then added to cells. The cells stained with a human IgG-Fc-specific pAb. 1 of 2 equivalent experiments is shown, again acquiring 30,000 events per sample.

We used the same ORF4 truncations as in Figure 2 to identify the GAG-binding domain of gp70, substituting a C-terminal IgG_1_ Fc domain for each GPI anchor (Figure 5). The relevant plasmids were transfected into 293T cells. Supernatants were harvested 48 h later and assayed for IgG Fc content (Figure 5A). Gp150 amino acids 1-151 was used as a control. SCR1-Fc showed minimal binding to BHK-21 and NMuMG cell surfaces (Figure 5B), but all the other ORF4 constructs bound well. Thus, SCRs1+2 were sufficient for cell binding, consistent with findings for the KSHV ORF4 [21]. The gp150 construct failed to bind.

The binding of the ORF4-Fc constructs was inhibited by soluble heparin (Figure 5C). The full-length construct was the least susceptible to inhibition, possibly reflecting multimerization. All of the constructs containing SCRs1+2 bound much better to GAG^+^ CHO cells than to the GAG-deficient CHO cell mutant pgs745 (Figure 5D). This confirmed GAG binding by gp70 and argued against the existence of an additional cell surface ligand. The SCR1-specific mAb T2B11 inhibited cell binding (Figure 5E), consistent with a dominant role for SCRs1-2.

### No evidence for GAG binding by the MHV-68 gB

**Figure 6:**
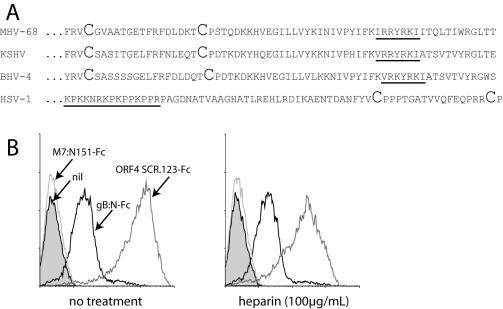
Heparin-independent binding by the N-terminal fragment of gB. A. Comparison of the gB regions containing the putative heparin binding motif of 3 gamma-herpesviruses-MHV-68, KSHV and BHV-4-and HSV-1. The first 2 conserved cysteine residues of each gB are shown in large type. B. Binding of Fc fusion proteins to NMuMG cells, pre-incubating or not (no treatment) the fusion protein with soluble heparin. gB:N-Fc is the first 423 amino acid residues of gB fused to human IgG_1_ Fc. 1 of 3 equivalent experiments is shown.

The gBs of HSV, KSHV and BHV-4 have all been reported to bind to GAGs [2], [6], [11]. The MHV-68 gB shares with these a run of cationic amino acids-considered a GAG binding motif-near its N-terminus. However, the motif in HSV is N-terminal to the first 2 conserved gB cysteine residues, whereas in the gammaherpesviruses it is C-terminal (Figure 6A). A recent crystal structure of the HSV-1 gB [25] allows computer-based homology modelling of other herpesvirus gBs, since their basic form is conserved. Homology modelling placed the GAG binding motif of MHV-68 (and that of other gammaherpesviruses) at the base of gB rather than near its crown (data not shown). The cationic residues were also buried. They therefore seem unlikely to interact with GAGs. Also, mutating 2 of these residues to alanines completely abrogates MHV-68 infectivity (L. Gillet and P.G. Stevenson, unpublished data), which would be more consistent with a major disruption of gB folding than with the loss of a superficial GAG binding site.

We tested recombinant gB for GAG binding by expressing its N-terminal domain (up to the furin-like cleavage site) as a fusion with IgG_1_-Fc (Figure 6B). The fusion protein bound to cells independently of heparin (Figure 6B). Cell binding by ORF4 SCRs 1-3 is shown for comparison, with a 5–10 fold inhibition of binding by heparin, consistent with Figure 5C. Thus, in contrast to descriptions of the KSHV and BHV-4 gBs, we found no evidence that the MHV-68 gB binds to GAGs.

### Recombinant gp150 can bind to GAGs

Gp150 amino acid residues 1-151 fused to IgG_1_-Fc failed to bind to cell surfaces (Figure 5B), as did fusions of residues 1-250 and 1-450 (data not shown). (The complete gp150 extracellular domain is 460 amino acids.) Nor was gp150 precipitated by heparin-agarose (Figure 4). However, the striking loss of MHV-68 GAG-dependence when gp150 is disrupted [20] strongly suggests that gp150 and GAGs somehow interact. It was possible that we had not observed a physical interaction because it was weak. We therefore expressed gp150 as a fusion with GST in *E.coli* to look further at cell binding. Gp150 has a predicted signal sequence of 22 amino acids. Residues 21-460 expressed very poorly with extensive C-terminal protein degradation (data not shown). We therefore focussed on the region 21-151. This contains many of the cationic residues in gp150, most of which is predicted to be highly acidic and heavily O-glycosylated. There are 3 cationic residues in the region 41–81 and 4 in 81–151.

**Figure 7:**
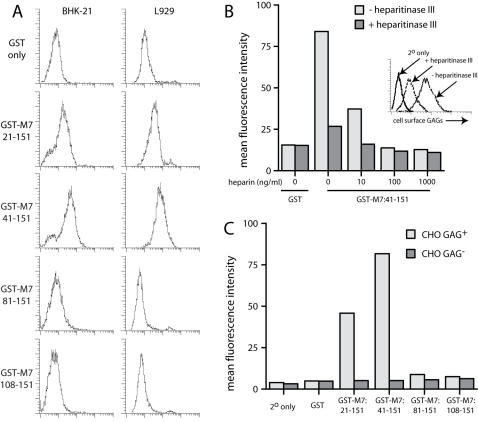
GAG binding by gp150 truncations fused to GST. A. BHK-21 or L929 cells were incubated with GST alone or GST fused to gp150 ( = M7) amino acid residues 21-151, 41-151, 81-151 or 108-151. Binding was detected with a biotinylated anti-GST pAb plus phycoerythrin-conjugated streptavidin and analyzed by flow cytometry. 1 of 5 equivalent experiments is shown. B. The GST-M7:41-151 fusion was preincubated with soluble heparin as shown before staining BHK-21 cells. These cells were pre-digested or not with heparitinase III. The effect of heparitinase III treatment on cell surface GAGs is shown in the inset. Protein binding was analyzed by flow cytometry as in A. 1 of 3 equivalent experiments is shown. C. Binding of the different GST-M7 fusions to GAG^+^ and GAG^−^ CHO cells was quantitated by flow cytometry. 1 of 3 equivalent experiments is shown.

Each GST fusion protein was recognized in ELISAs by at least 5 different gp150-specific mAbs (data not shown). Thus, the native conformation of each gp150 fragment appeared to be preserved. GST-M7:21-151 and GST-M7:41-151 both bound to BHK-21 and L929 cells, whereas GST-M7:81-151 and GST-M7:108-151 did not (Figure 7A). Convincing binding required 50 µg/ml of fusion protein; at 10 µg/ml the binding was minimal (data not shown). The binding was inhibited by soluble heparin and by heparitinase III treatment of the cells (Figure 7B). GST-M7:21-151 and GST-M7:41-151 also bound to GAG^+^ but not GAG^−^ CHO cells (Figure 7C), whereas GST-M7:81-151 and GST-M7:108-151 bound to neither. Thus, an N-terminal region of gp150 was capable of GAG binding. The better binding of GST-M7:41-151 relative to GST-M7:21-151 suggested that residues 21-41 might inhibit GAG binding.

### ORF4-deficient MHV-68 remains heparin-sensitive

**Figure 8:**
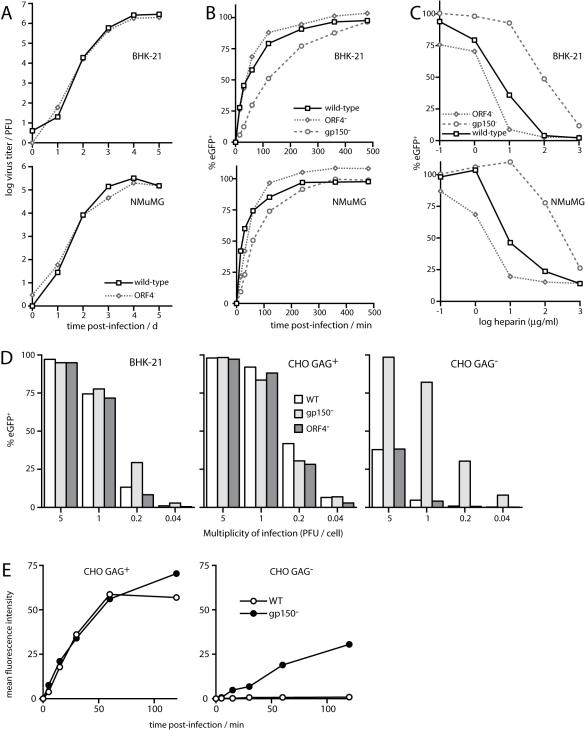
Infectivity of gp150 and gp70 MHV-68 knockout viruses. A. Wild-type and ORF4-deficient (ORF4^−^) viruses were tested for growth in BHK-21 and NMuMG cells after low multiplicity infection (0.01 PFU/cell). The data are from 1 of 2 equivalent experiments. B. BHK-21 or NMuMG cells were exposed to wild-type, ORF4-deficient (ORF4^−^) or gp150-deficient (gp150^−^) virions for different times before washing with PBS. 18 h later viral eGFP expression was assayed by flow cytometry. Each value is expressed as a percentage of the eGFP expression of unwashed cells. The data are from 1 of 2 equivalent experiments. C. Wild-type, ORF4-deficient (ORF4^−^) and gp150-deficient (gp150^−^) virions were preincubated with heparin then added to BHK-21 or NMuMG cells. 18 h later, viral eGFP expression was assayed by flow cytometry. Each value is expressed as a percentage of the eGFP expression with untreated virus. The data are from 1 of 2 equivalent experiments. D. BHK-21, CHO GAG^+^ or CHO GAG^−^ cells were infected overnight with wild-type (WT), gp150-deficient (gp150^−^) or ORF4-deficient viruses (ORF4^−^) in the presence of 10 µg/ml phosphonoacetic acid to inhibit any viral spread. Each virus expressed eGFP from an HCMV IE1 promoter in the BAC cassette at the left end of the genome. The number of infected cells was assessed by flow cytometric counting of eGFP^+^ cells. The data are from 1 of 2 equivalent experiments. E. CHO GAG^+^ or CHO GAG^−^ cells were exposed to gp150^+^ (WT) or gp150^−^ virions that were fluorescent by virtue of an eGFP tag on glycoprotein M. At the times shown, the cells were washed with PBS to remove unbound virions. Virion binding was then quantitated by flow cytometry. The data are from 1 of 2 equivalent experiments.

We reasoned that if the GAG dependence of wild-type MHV-68 infection [20] reflected an important contribution of gp70-the major MHV-68 heparin binding protein-then a gp70-deficient mutant should show an infectivity deficit and resist further inhibition by heparin. Neither was the case (Figure 8). ORF4-deficient MHV-68 showed no significant deficit in growth (Figure 8A) or cell binding (Figure 8B) compared to wild-type and was more sensitive to inhibition by soluble heparin rather than less (Figure 8C). By contrast, gp150-deficient MHV-68 had a small deficit in binding to GAG^+^ cells (Figure 8B) but was largely resistant to heparin (Figure 8C).

ORF4-deficient MHV-68 was further equivalent to wild-type in its infection of GAG^+^ and GAG^−^ CHO cells, whereas gp150-deficient MHV-68 infected GAG^−^ CHO cells much better (Figure 8D). This increased infection appeared to reflect better cell binding (Figure 8E). Since the weak interaction of gp150 with GAGs had much greater function consequences for MHV-68 infection than the strong interaction of gp70, we conclude that GAGs do not promote infection by providing strong binding themselves, but by reversing a constitutive inhibition of cell binding imposed by gp150.

## Discussion

GAGs are generally thought to promote herpesvirus infections by adsorbing virions to cell surfaces, thereby increasing their chance of engaging key protein ligands. The interaction between MHV-68 and GAGs appeared to be different. Gp70 bound strongly to GAGs but played little direct role in infection, whereas gp150 bound at best weakly, yet was the major determinant of GAG dependence. The lack of a gp70-related infection deficit could conceivably reflect that another virion glycoprotein also binds strongly to GAGs, but the weak binding of gp150 seemed unlikely to fulfill such a role. Moreover, the main effect of removing gp150 was not reduced binding to GAG^+^ surfaces, but increased binding to and infection of GAG-deficient cells. We interpret these findings as MHV-68 using GAGs as a gp150-dependent entry signal, rather than a significant physical attachment.

Herpes virions are inevitably exposed to antibody when they exit immune hosts. Their survival therefore depends on protecting key glycoprotein epitopes. They do this without recourse to antigenic variation or generalized immune suppression. Protection is likely to depend instead on mechanisms akin to the sequential uncoating proposed for HIV [26]. The greater transmissibility of herpesviruses implies that their evasion is even more effective. Indeed, antibody evasion provides a plausible rational for the whole multi-protein complexity of herpesvirus entry. HSV-1 currently provides the most detailed picture of how this might work at a molecular level, with glycoprotein D changing conformation on ligand binding to activate the gB/gH/gL fusion complex [27]–[29]. We hypothesize that GAGs initiate equivalent conformational changes in gp150. Such a mechanism would not necessarily require strong GAG binding, particularly if ligands such as gH/gL [30] also provide attachment. Thus, gp150 could provide a shield against antibody that is displaced only at the plasma membrane.

Cells shed GAGs in response to a variety of stimuli, including inflammation [1]. MHV-68 must therefore protect its entry mechanism against premature activation by soluble GAGs, which would expose it to neutralizing antibody. Weak GAG binding may achive this by making gp150 interactions reversible unless a second ligand is subsequently engaged. Such an arrangement would be consistent with soluble heparin competitively antagonizing entry rather than promoting it. The abundantly shed gp45/gp55 heparin-binding SCRs of ORF4 could also provide protection by locally absorbing soluble GAGs, in addition to saturating cell-associated GAGs to reduce virion re-capture. The increased sensitivity of ORF4-deficient virions to inhibition by soluble heparin-here SCR shedding should not have been a significant factor-suggested that gp70 may also improve the selectivity of gp150 for membrane-bound GAGs. Thus, through gp150 and gp70 MHV-68 can both exploit GAGs as an entry signal and cope with the attendant problems of GAG shedding and virion release.

Since GAGs are important for MHV-68 infection, it might be expected that antibodies could neutralize virions by blocking GAG binding. However, we have not yet found any mAbs capable of doing so [31], [32]. In fact, gp150-specific mAbs generally improved cell binding by the M7:21-151 and M7:41-151 GST fusions, presumably by increasing their avidity (data not shown). The only gp70-specific mAb that inhibited GAG binding, T2B11, was an IgM, which may therefore act by steric hindrance rather than by binding to the GAG interaction site itself. It did not neutralize virions (data not shown). One reason why GAG interactions might be hard to block was suggested by the binding of the ORF75c, ORF75b and ORF65 gene products to heparin-agarose (Figure 4). If nuclear localization signals and nucleic acid binding sites can comprise cationic patches sufficient to bind GAGs, antibodies directed against such patches would be potentially auto-reactive and therefore strongly selected against. GAG binding is also a feature of some important cellular growth factors. Thus, GAGs may provide a relatively antibody-resistant interaction which viruses exploit to exit and enter immune hosts.

## Material and Methods

### Monoclonal antibodies

Female BALB/c mice were were infected intranasally with MHV-68 (2×10^4^) in accordance with Home Office Project Licence 80/1579. A 10^7^ PFU intraperitoneal boost was given 3–4 months later. Spleens were harvested after a further 3 days. Splenocytes were fused with NS0 cells and selected with azaserine (1 µg/ml)/hypoxanthine (100 µM) [33]. MAbs were concentrated from tissue culture supernatant by ammonium sulfate precipitation and quantitated by ELISA using isotype-matched standards. MAbs against gB (T7H9) [23], gp150 (T1A1) [20], gN (3F7) [33] and gH/gL (T4C5) [30] have been described.

### Viruses

ORF4-deficient [34] and gp150-deficient [20] MHV-68 mutants, and MHV-68 with eGFP-tagged glycoprotein M [32] have been described. The gp150 STOP mutation [20] was transferred onto the eGFP-tagged gM background by BAC-based mutagenesis in *E.coli* [34]. Virus stocks were grown and titered by plaque assay in BHK-21 cells [35]. Infected cells and supernatants were sonicated after harvesting, cell debris was pelleted by low-speed centrifugation (1000×g, 3 min), and virions were recovered from supernatants by high speed centrifugation (38,000×g, 90 min). To prepare purified virions, the pelleted virions were centrifuged on 5–15% Ficoll gradients (30,000×g, 90 min), recovered as a distinct band, pelleted (30,000×g, 90 min), sonicated, and stored at −70°C [36]. All viruses used in infectivity experiments were plaque-assayed in parallel. Equivalent protein content was confirmed by immunoblotting an equivalent number of PFU for the capsid components encoded by ORF17 (data not shown).

### Plasmids

The full-length ORF4 coding sequence (genomic co-ordinates 9873-11039) [37] was amplified by PCR (Hi-Fidelity PCR kit, Roche Diagnostics Ltd), including *Eco*RI and *Xho*I restriction sites in the respective 5′ and 3′primers, and cloned into the *Eco*RI/*Xho*I sites of pMSCV-IRES-ZEO [38]. ORF4 truncations incorporating short consensus repeat (SCR) 1 (amino acid residues 1-82), SCRs 1+2 (amino acids 1-151), SCRs 1+2+3 (amino acids 1-209), SCRs 1+2+3+4 (amino acids 1-270), and SCRs 1+2+3+4 and the ST domain (amino acids 1-354) were amplified with 5′ *Avr*II-restricted and 3′ *Not*I-restricted primers and cloned into the *Xba*I/*Not*I sites of pBRAD [23] to attach a C-terminal glycosyl-phosphatidyl-inositol (GPI) membrane anchor, or into the same sites of pTORSTEN [23] to attach human IgG_1_-Fc. The coding sequence for amino acid residues 1-151 of gp150 was amplified with 5′ *Xba*I-restricted and 3′ *Not*I-restricted primers and cloned into the same sites of pTORSTEN. To make GST fusions of gp150, the relevant portions of its coding sequence (amino acid residues 21-151, 41-151, 81-151 and 108-151 were amplified by PCR with *Eco*RI-restricted and *Xho*I-restricted 5′ and 3′ primers respectively. The 3′ primer also included a 6×histidine tag. The PCR products were cloned into the *Eco*RI and *Xho*I sites of pGEX-4T-1 (APBiotech, Little Chalfont, U.K.) so as to place the gp150 fragments downstream of and in frame with GST.

### Protein expression in *E.coli*

Plasmids derived from pGEX-4T1 were transformed into BL-21 *E.coli*. Expression was induced for 4 h in log phase growth with 400 µM IPTG. The *E.coli* were lysed in 1% Triton X-100/50 mM TrisCl pH 7.4/150 mM NaCl/5 mM EDTA/1 mM PMSF/5 mM N-ethyl-maleimide/Complete protease inhibitors (Roche Diagnostics). The GST fusion proteins were precipitated with glutathione-sepharose beads (APBiotech), washed×3 in lysis buffer and×2 in Tris-saline, eluted with 50 mM glutathione, and dialysed×3 against PBS.

### Cells

Baby Hamster Kidney cells (BHK-21), L929 cells, NMuMG cells, CHO-K1 cells, the GAG-deficient CHO-K1 mutant pgs745, and 293T cells were all grown in Dulbecco's modified Eagle medium (Invitrogen, Paisley, U.K.) supplemented with 2 mM glutamine, 100 U/ml penicillin, 100 µg/ml streptomycin and 10% fetal calf serum (PAA laboratories, Linz, Austria). CHO-K1 cells expressing ORF4 were generated by transfection with pMSCV-ORF4-IRES-ZEO using Fugene-6 (Roche Diagnostics Ltd., Lewes, U.K.) and selected in 500 µg/ml Zeocin (Invitrogen Corporation, Paisley, U.K.). 293T cells were also transfected using Fugene-6.

### Immunoprecipitation and Immunoblotting

BHK-21 cells were infected with MHV-68 (2 PFU/cell, 18 h), starved for 45 min in cysteine/methionine-free RPMI (Sigma Chemical Co., Poole, U.K.), then labelled for 1 h with ^35^S-cysteine/methionine (PerkinElmer Life Sciences, Cambridge, U.K.) [38]. The cells were lysed on ice for 30 min in 1% Triton X-100, 50 mM TrisCl pH 7.4, 150 mM NaCl with 1 mM PMSF and Complete protease inhibitors (Roche Diagnostics). Insoluble debris was pelleted by centrifugation (13,000×g, 15 min). The lysates were then pre-cleared with protein A-sepharose and immunoprecipitated with monoclonal antibody plus further protein-A-sepharose. The agarose beads were washed×5 in lysis buffer and heated (95°C, 5 min) in Laemmli's buffer. The denatured proteins were resolved by SDS-PAGE. Dried gels were exposed to X-ray film. To immunoprecipitate viral proteins with heparin-agarose, virions were recovered from infected BHK-21 cell supernatants by ultracentrifugation (38000×g, 90 min), then lysed as above. The lysates were incubated with heparin-agarose beads (Sigma Chemical Co.), with or without 1 mg/ml soluble heparin. The beads were washed×5 in lysis buffer, and the attached proteins analysed by SDS-PAGE. For total protein staining, gels were fixed in 10% acetic acid/50% methanol and stained with Coomassie R250. After destaining, bands of interest were excised and analysed by MALDI fingerprinting at the Protein and Nucleic Acids Centre, Department of Biochemistry, Cambridge. For immunoblotting [39], the proteins were transferred to PVDF membranes (Perbio Science, Tattenhall, U.K.). The membranes were blocked with 10% non-fat milk in PBS/0.1% Tween-20, then incubated with MHV-68 glycoprotein-specific mAbs or with an MHV-68-immune rabbit serum [40]. Bound antibody was detected with horseradish peroxidase-conjugated rabbit anti-mouse IgG pAb (Dako Corporation, Ely, U.K.) or horseradish peroxidase-conjugated donkey anti-rabbit IgG pAb (APBiotech), followed by washing in PBS/0.1% Tween-20, development with ECL substrate (APBiotech) and exposure to X-ray film. Immunoblotting of secreted proteins or virion proteins followed a similar protocol. IgG_1_-Fc fusion proteins were detected with a horseradish peroxidase-conjugated goat anti-human IgG-Fc pAb (Sigma Chemical Co.). Where indicated, proteins were deglycosylated before immunoblotting with PNGase F or sialidase+O-glycanase (Prozyme, San Leandro, CA).

### Flow cytometry and immunofluorescence

Cells were incubated with glycoprotein-specific mAbs (1 h, 4°C), washed×2 in PBS, incubated with fluorescein-conjugated rabbit anti-mouse IgG pAb (Dako Cytomation, Ely, U.K.), washed×2, and analysed on a FACS Calibur (BD Biosciences, Oxford, U.K.). The binding of Fc fusion proteins was detected with a phycoerythrin-conjugated goat anti-human IgG-Fc pAb (Sigma Chemical Co.). The binding of GST fusion proteins was detected with a biotinylated goat anti-GST pAb (Santa Cruz Biochemicals, Santa Cruz, CA) followed by phycoerythrin-conjugated streptavidin (BD Biosciences). Graphs were plotted with FCSPress v1.3.
